# Certification of NIST Room Temperature Low-Energy and High-Energy Charpy Verification Specimens

**DOI:** 10.6028/jres.120.020

**Published:** 2015-12-03

**Authors:** Enrico Lucon, Chris N. McCowan, Ray L. Santoyo

**Affiliations:** National Institute of Standards and Technology, Boulder, CO 80305

**Keywords:** Charpy master machines, Charpy reference specimens, coefficient of variation, high-energy specimens, indirect verification, low-energy specimens, room temperature, sample size, specimen jamming

## Abstract

The possibility for NIST to certify Charpy reference specimens for testing at room temperature (21 °C ± 1 °C) instead of −40 °C was investigated by performing 130 room-temperature tests from five low-energy and four high-energy lots of steel on the three master Charpy machines located in Boulder, CO. The statistical analyses performed show that in most cases the variability of results (i.e., the experimental scatter) is reduced when testing at room temperature. For eight out of the nine lots considered, the observed variability was lower at 21 °C than at −40 °C. The results of this study will allow NIST to satisfy requests for room-temperature Charpy verification specimens that have been received from customers for several years: testing at 21 °C removes from the verification process the operator’s skill in transferring the specimen in a timely fashion from the cooling bath to the impact position, and puts the focus back on the machine performance. For NIST, it also reduces the time and cost for certifying new verification lots. For one of the low-energy lots tested with a C-shaped hammer, we experienced two specimens jamming, which yielded unusually high values of absorbed energy. For both specimens, the signs of jamming were clearly visible. For all the low-energy lots investigated, jamming is slightly more likely to occur at 21 °C than at −40 °C, since at room temperature low-energy samples tend to remain in the test area after impact rather than exiting in the opposite direction of the pendulum swing. In the evaluation of a verification set, any jammed specimen should be removed from the analyses.

## 1. Introduction

Charpy impact testing is frequently specified as an acceptance test for structural materials, and all laboratories performing acceptance tests are expected to periodically verify the performance of their Charpy impact machine(s). According to the ASTM E23-12c standard [1], the procedure for verifying the performance of Charpy machines consists of a direct verification and an indirect verification.

The direct verification corresponds to a detailed evaluation of the machine dimensions, alignment, etc., while the indirect verification of the machine performance is carried out by breaking sets of Charpy reference specimens with certified values of absorbed energy. The indirect verification procedure was added to ASTM E23 more than 50 years ago, when it was ascertained that direct verification alone could not explain certain unacceptable differences (as much as 100 %) among the results of the machines tested. Since some of the differences originated from interactions between the machine components and the specimens, only actual Charpy tests on reference specimens could resolve these effects [2].

Currently and for the last 26 years, NIST in Boulder has supplied impact reference specimens as a Standard Reference Material (SRM), which is used to indirectly verify the performance of Charpy machines in accordance with ASTM E23. Historically, the Charpy verification program was developed by the U.S. Army (Watertown Arsenal, AMMRC) that produced and distributed reference specimens for the verification of Charpy machines in the United States. The Army procedures were adopted by ASTM in their E23 standard in 1956 (ASTM E23-56T). As a result of the adoption of the E23 procedures and requirements, the differences between the Charpy machines of the Army contractors were reduced to 1 ft-lb (1.4 J) or 5 %, whichever was greater [2].

The Charpy verification program was taken over by NIST in 1989, and Army personnel helped to transfer the reference Charpy machines and their evaluation procedures to NIST. The three reference Charpy machines have been defined in ASTM E23 as the “master Charpy impact machines” for 25 years [3]. Each year, the NIST program evaluates the indirect verification test results of over 1500 industrial machines. If the test results of an industrial machine agree with the average results of the NIST master machines within 1.4 J or 5 %, whichever is greater, the machine is certified for acceptance testing according to the requirements of ASTM Standard E23.

Besides NIST, there are four other institutes in the world that certify and distribute reference Charpy specimens for the indirect verification of impact machines:

- The *Joint Research Center Institute for Reference Materials and Measurements* (JRC-IRMM) of the European Commission, located in Geel (Belgium). Their Charpy verification specimens cover four levels of absorbed energy, corresponding approximately to 25 J, 80 J, 120 J, and 150 J. All specimens must be tested at room temperature (RT, 20 °C), even though one batch of low-energy specimens also has certified values at 0 °C to avoid jamming [4]. Tests are performed and evaluated in accordance with ISO 148-2:2008 [5], and under ISO/IEC 17025 accreditation. The production of Charpy reference materials is covered by JRC-IRMM’s ISO Guide 34 accreditation.
- *Laboratoire National de Métrologie et d’Essais* (LNE), located in Trappes near Paris (France). Their reference specimens cover five absorbed energy levels, namely: low (approx. 25 J), medium (70 J to 80 J), high 1 (115 J to 125 J), high 2 (160 J to 175 J), and super high (200 J to 220 J). All specimens have to be tested at room temperature (20 °C) in accordance with ISO 148-2:2008.
- *Institut für Eignungsprüfung* (IfEP), located in Marl (Germany). They provide certified reference Charpy specimens at four absorbed energy levels (low, 15 J − 39 J; medium, 40 J − 99 J; high, 100 J − 159 J; super-high, 160 J − 200 J+). Tests are performed at room temperature in accordance with ISO 148-2 or ASTM E23.
- The *National Metrology Institute of Japan* (NMIJ), located in Tsukuba (Japan). NMIJ (then called National Research Laboratory of Metrology, NRLM) used to certify and distribute Charpy reference specimens of steel corresponding to different absorbed energy levels [6]. However, a recent internet search has shown that at the time of writing, only reference materials for the Charpy impact strength of plastics (PVC and PMMA) are available from NMIJ [7].

To the authors’ knowledge, similar Charpy verification programs are expected to be launched soon by other international institutes, such as the Shanghai Research Institute of Materials (SRIM, China), the National Institute of Metrology, Quality and Technology (Inmetro, Brazil), and the National Physical Laboratory (CSIR-NPL, India).

Since the time the U.S. Charpy verification program was run by the Army, verification specimens have to be tested at −40 °C (−40 °F) for the levels corresponding to low energy (14 J − 20 J at −40 °C, SRM 2092) and high energy (88 J − 136 J at −40 °C, SRM 2096). Initially, only these two energy levels were available. With the development of new steels that have higher toughness and impact strength, a third absorbed energy level (super-high energy, approximately 200 J) was introduced in the mid-90s, following customers’ demand. Super-high-energy verification specimens made from an 18 Ni, cobalt-strengthened maraging steel designated as T-200, were certified at NIST for testing at room temperature (21 °C ± 1 °C). For many years, NIST has been approached by customers with requests and discussions on the possibility of certifying Charpy specimens for low- and high-energy verification at room temperature instead of −40 °C. If the test is performed at room temperature, the operator’s skill in transferring the specimen from the temperature bath in less than 5 seconds is removed from the verification test. The same applies for other ancillary experimental components, such as the accuracy and the calibration state of the temperature-measuring equipment. Therefore, it can be argued that the focus of the verification test is solely on the machine performance. Room-temperature SRMs have two additional advantages:

- for the customer, the need to invest in cooling-bath equipment is removed, if not required for general testing;
- for NIST, the time and cost for the certification of a room-temperature lot is significantly reduced with respect to a −40 °C lot.

The feasibility study described in this paper was aimed at evaluating the possibility of providing NIST customers with the option of conducting their verification tests at room temperature (21 °C ± 1 °C) rather than at −40 °C. As detailed above, this would put NIST in line with the remaining current producers of Charpy verification specimens (NMIs), with the exception of the 0 °C, low-energy batch provided by IRMM.

To justify this change and satisfy customers’ requests, we evaluated the influence that testing at RT would have on the variation (scatter) of several batches of both low- and high-energy Charpy reference specimens. If the variation can be matched or even reduced, RT Charpy verification specimens could (and should) be produced and made available to the NIST customer base.

## 2. Material, Test Equipment, and Test Matrix

Both the low- and high-energy SRMs are made from AISI 4340 steel bars from a single heat to minimize compositional and microstructural variations. The nominal composition of the 4340 steel is presented in Table 1.

The steel is produced by a double-vacuum-melting procedure (vacuum-induction-melt and vacuum-arc-remelt), in order to minimize elements such as P, S, Va, Nb, Ti, and Cu.

Ingots are forged, hot-rolled, and cold-finished to 12.7 mm square bars, and finally annealed. The maximum acceptable grain size is ASTM #8. The bars are then normalized at 950 °C and hardened to approximately 35 HRC (Rockwell Hardness C).

To produce different levels of Charpy absorbed energy, the steel is heat-treated by tempering for 1.5 h between 300 °C and 400 °C for low-energy specimens, and for 1.25 h at 593 °C for high-energy specimens.

Additional details on specimen production, sampling and machining are available in [3].

The Charpy machines used in this study are the three master machines located at NIST in Boulder, Colorado. Their principal characteristics are listed in Table 2.

For this study, we tested specimens from nine lots of verification specimens: five at the low-energy level (LL) and four at the high-energy level (HH). Of these nine lots, one (HH-149) was a “failed” lot, i.e., rejected for use as verification specimens at −40 °C, based on a sample size greater than five^1^.

Two of the lots were tested on all three master machines (typically, 25 tests per machine); each of the remaining seven batches were tested on one machine only (again, typically 25 tests). All tests were performed at room temperature (21 °C ± 1 °C).

The complete test matrix is presented in Table 3.

## 3. Statistical Analyses

The statistics listed below are returned when Charpy test results from a single machine or multiple machines are analyzed.

### 3.1 Test Result Statistics

– Number of tests performed (*N*).
– Mean value of absorbed energy $\left(\bar{KV}\right)$:
  $$\bar{KV}=\frac{1}{N}\sum_{i=1}^{N}KV_{i}$$ (1)
  where *KV_i_* is the value of absorbed energy (in J) obtained from the *i*-th test, with *i* = 1,…,*N.* − Standard deviation (σ*_KV_*):
  $$\sigma_{KV}=\sqrt{\frac{1}{N}\sum_{i=1}^{N}\left(KV_{i}-\bar{KV}\right)}$$ (2)
– Variance $\left(\sigma_{KV}^{2}\right)$:
  $$\sigma_{KV}^{2}=\frac{1}{N}\sum_{i=1}^{N}{\left(KV_{i}-\bar{KV}\right)}^{2}$$ (3)
– Degrees of Freedom (*v*):
  v=N−1 (4)
– Experimental standard deviation of the mean $\left(SE_{\bar{KV}}\right)$:
  $$SE_{\bar{KV}}=\frac{\sigma_{KV}}{\sqrt{N}}$$ (5)
– Smallest (*KV*_min_) and largest (*KV*_max_) value of absorbed energy.
– Range of absorbed energy values (*KV*_max_ – *KV*_min_).
– Coefficient of variation (*CV*):
  $$CV=\frac{\sigma_{KV}}{\bar{KV}}$$ (6)

### 3.2 Machine Statistics

The same statistics listed under Sec. 3.1 are individually outputted for each of the impact machines used.

### 3.3 Additional Statistics

- Equality of variances: the hypothesis that the machine variances are equal is verified by means of Levene’s test [8]. The output of the test is a *p-value*. If this is lower than the critical *p-value* corresponding to a significance value *α* = 0.05, the assumption of equal variances is rejected and the observed differences in sample variances are unlikely to have occurred based on random sampling from a population with equal variances.
- Pooled standard deviation: in statistics, pooled variance is a method for estimating the variance of several different populations when the mean of each population may be different, but one may assume that the variance of each population is the same [9]. The square root of a pooled variance is known as a pooled standard deviation (*s*_p_). It accounts for possibly different sample sizes for each machine, and for the three NIST master machines is given by:
  $$s_{p}=\sqrt{\frac{s_{1}^{2}+s_{2}^{2}+s_{3}^{2}}{3}}$$ (7)
  where *s*_1_, *s*_2_, and *s*_3_ indicate respectively the standard deviations of the three master machines.
- ASTM Pass/Fail: firstly, the deviation between the mean of each machine and the grand mean (mean of the means for each machine) is calculated. If the deviation is less than 1.4 J or 5 % of the grand mean (whichever is larger), the machine passes the ASTM E23 criterion. Additionally, the *k-ratio* is calculated for each machine, by dividing the machine’s standard deviation by the pooled standard deviation. The *k-ratio* should be less than 1.25, based on 3 machines and 25 measurements per machine [3,10]. If any of the *k-ratio* values is greater than 1.25, the variability in energy values attributable to that machine is questionable and appropriate actions should be taken (direct verification, repairs, testing of additional specimens, etc.).
- Sample size: this represents the minimum number of specimens from a given production lot that should be tested in a verification test. It is a very important statistical metric for assessing the quality of a reference specimen lot. It is defined as [3,11]:
  $$n_{ss}={\left(\frac{3s_{p}}{E}\right)}^{2},$$ (8)
  where *E* is 1.4 J or 5 % of the grand mean, whichever is greater. The sample size is one of the statistics used to determine the acceptability of a lot^2^ and the performance of the machines.
- Maximum *s*_p_: for low-energy specimens (*E* = 1.4 J), the maximum pooled standard deviation allowed for a sample size of 5 is given by:
  $$\mathrm{Max}\;s_{p}=1.4\frac{\sqrt{5}}{3}=1.043\;J;$$ (9)
  for high-energy specimens $\left(E=0.05\;\bar{KV_{\text{gm}}}\right)$, it is given by:
  $$\mathrm{Max}\;s_{p}=0.05\frac{\sqrt{5}}{3}\bar{KV_{\text{gm}}}=0.037\cdot \bar{KV_{\text{gm}}},$$ (10)
  where $\bar{KV_{\text{gm}}}$ is the grand mean of the test results. Equation. (9) and Eq. (10) can be obtained from Eq. (7) for *n*_SS_ = 5.

Obviously, the statistics listed in (a–c) above are only meaningful when tests are performed on more than one machine.

### 3.4 Criteria for Assessing the Feasibility of RT Verification Specimens

In this study, two statistical parameters are primarily used to characterize the variability (scatter) of Charpy results, and hence to assess the feasibility of producing NIST verification specimens to be tested at room temperature:

– the coefficient of variation *CV*, Eq. (6), and
– the sample size *n*_SS_, Eq. (8).

If both *CV* and *n_ss_* calculated from room temperature tests are lower than or equivalent^3^ to the values obtained at −40 °C under the same experimental conditions (same machine(s) and approximately the same number of tests), the feasibility is demonstrated for a particular specimen lot.

## 4. Results

The detailed results of the tests performed are reported in the NIST Internal Report 8087 [12], which is publicly available.

### 4.1 Lots Tested on One of the Master Machines

The results obtained on low-energy and high-energy Charpy lots tested on a single master machine are summarized in Table 4 and compared to the results previously obtained at −40 °C (pilot and production lots combined). In all cases, the results obtained at 21 °C were better then at −40 °C (lower values of *CV* and *n_SS_*).

### 4.2 Lots Tested on All Three Master Machines

The results obtained on the two lots tested on all three master machines (SI, TK, and TO) are shown in Table 5 and compared to the results previously obtained at −40 °C (pilot and production lots combined). The results obtained at room temperature are better for the low-energy batch and worse for the high-energy batch. However, this latter is a “failed” lot, which could not be certified due to a sample size greater than 5.0. Moreover, for HH-149 the value of *CV* at room temperature is only 7 % higher than its *CV* at −40 °C, while *n*_SS_ at RT is 20 % higher than at −40 °C.

## 5. Discussion

### 5.1 Relationships between Results at −40 °C and 21 °C

**Fig. 1:**
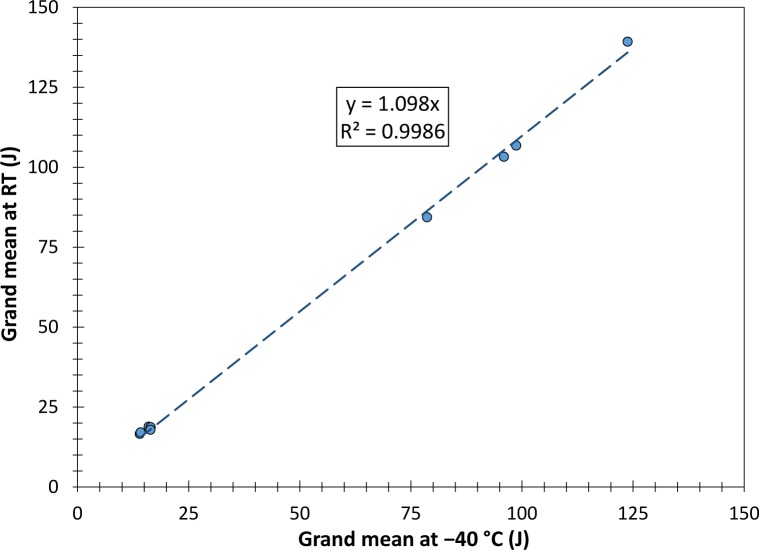
Relationship between absorbed energy (grand means) at room temperature and −40 °C.

The grand means obtained at 21 °C and −40 °C (average of pilot and production lot tests) are compared in Fig. 1 for every batch tested. Based on a linear fit, the absorbed energy values at room temperature are 10 % ± 4 % (95 % confidence) higher than at −40 °C.

**Fig. 2:**
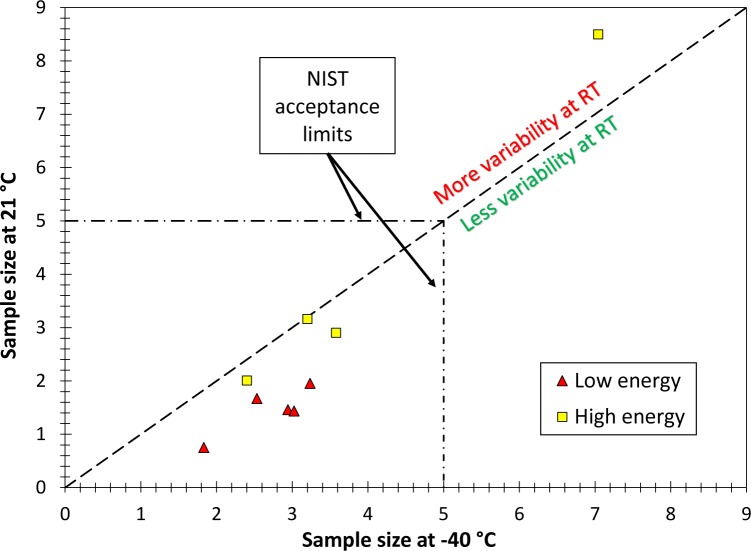
Relationship between sample size at room temperature and −40 °C.

A similar comparison is shown in Fig. 2 for the sample size *n*_SS_. In the figure, the upper left half corresponds to an increased variability at RT with respect to −40 °C, the lower right half to a reduced variability. Sample sizes at −40 °C were obtained by averaging the values calculated for the pilot lot and the production lot.

All lots examined in this study, with the exception of the “failed” lot HH-149, show lower variability (lower sample size) at 21 °C than at −40 °C. It’s interesting to note that the scatter reduction is more significant for low-energy specimens than for high-energy specimens.

### 5.2 Low-Energy Specimens Jammed

When low-energy verification specimens are tested at −40 °C, in most cases the broken specimens exit the machine in a direction opposite to the pendulum swing. This minimizes the chances of post-test secondary interactions between specimen halves and the swinging pendulum, or other parts of the machine (anvils, supports, shrouds if present).

However, when testing low-energy specimens at room temperature, we noticed that oftentimes the broken specimens are not ejected from the machine, but remain close to the test area because of the slightly higher impact toughness (around 10 % according to our results – see Fig. 1). As a consequence, secondary impacts with the swinging hammer become more frequent and the likelihood increases of one or both specimen halves jamming and dissipating pendulum energy.

**Fig. 3:**
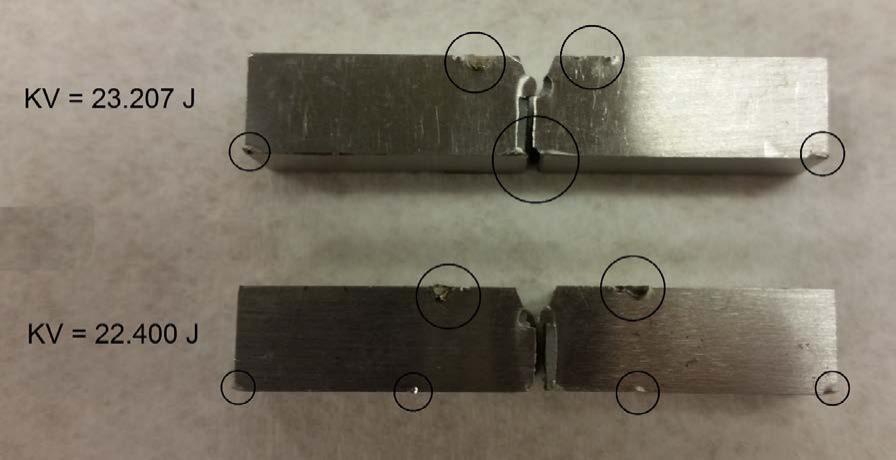
LL-133 outlier specimens, showing clear signs of jamming (circled).

Out of 130 low-energy specimens tested at room temperature, only two specimens (1.5 %) showed clear evidence of jamming, as indicated by both their significantly high *KV* values and the marks visible on the broken halves (Fig. 3). Both tests were performed on the same master machine (TK) and on the same low-energy batch (LL-133).

To confirm the anomalous nature of these test results from a statistical standpoint, we used a common statistical test for outlier detection: Grubbs’ test, also known as the maximum normed residual test or extreme studentized deviate test [13]. Both tests were identified as outliers:

- The highest *KV* value (23.21 J, compared to an average of 16.85 J for the remaining 29 tests) corresponded to a *Z-value* of 3.644, which was higher than the critical value of *Z* (2.908) at a significance level of 0.05.
- The second highest *KV* value (22.40 J, compared to an average of 16.65 J for the remaining 28 tests) corresponded to a *Z-value* of 4.459, which was higher than the critical value of *Z* (2.893) at a significance level of 0.05.

Grubbs’ test performed on the remaining 28 test results did not detect any residual outliers. The coefficient of variation dropped from 0.099 to 0.074 (first outlier removed) to 0.039 (second outlier removed); the sample size decreased from 13.078 to 7.127 (first outlier removed) to 1.956 (second outlier removed).

Even though the TK machine is the only master machine that has a C-shaped pendulum, a possible effect of machine design on the occurrence of specimen jamming can be ruled out, since it has been shown that specimen jamming can also occur on machines equipped with a U-shaped pendulum, if shrouds are absent or incorrectly positioned [14].

**Fig. 4:**
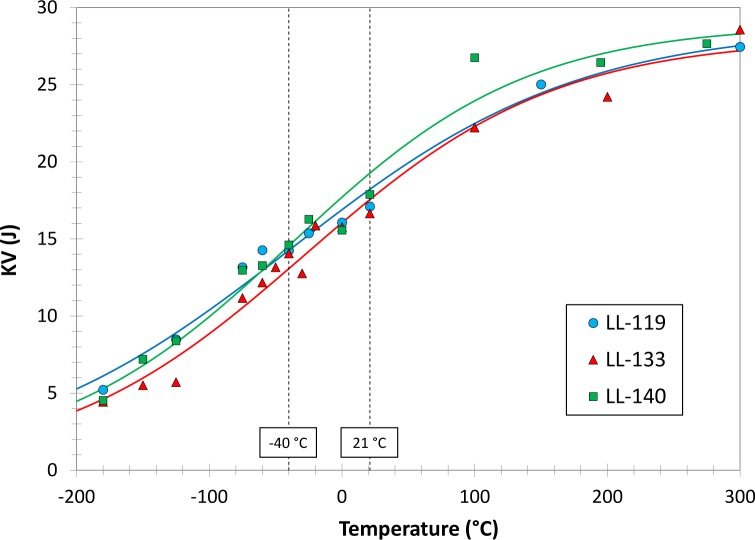
KV transition curves for LL-119, LL-133, and LL-140.

It is also interesting to note that jamming occurred only for LL-133, but not for the other two low-energy lots tested on the TK machine (LL-119 and LL-140). We therefore decided to compare the three low-energy lots in terms of full energy vs. temperature transition curves, obtained by fitting results between −180 °C and 300 °C with hyperbolic tangent regression curves. The comparison of the transition curves in Fig. 4 shows that the differences in absorbed energy among the three lots are negligible both at −40 °C and 21 °C.

**Fig. 5:**
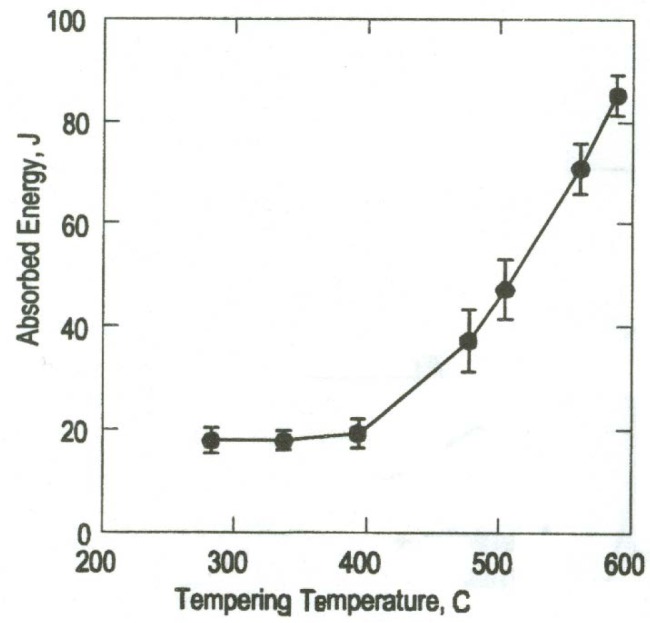
Effect of tempering temperature on-room temperature absorbed energy for 4340 steel [3].

It is uncertain, therefore, whether a modification of the heat treatment for the low-energy 4340 steel, such as lowering the tempering temperature below 400 °C or modifying the duration of the heat treatment, could effectively decrease the likelihood of jamming. Furthermore, the trend of absorbed energy as a function of tempering temperature for 4340 shown in Fig. 5 [3] indicates that *KV* is not very sensitive to tempering temperatures below 400 °C.

All things considered, the slightly higher likelihood of a low-energy specimen jamming at room temperature does not represent a serious hurdle for developing room temperature reference specimens. Even for specimens tested at −40 °C, the current NIST procedure calls for removing from the analyses any specimen showing evidence of jamming or other test-related issues (such as a specimen struck off-center or badly positioned). When a NIST customer sends back a sample that has clearly jammed and whose absorbed energy is significantly higher than the rest of the verification set, its result is ignored and the machine verification is based on the *KV* values from the remaining specimens.

### 5.3 Severity of Room-Temperature Verification at Low-Energy Level

An additional objection to the production of room-temperature low-energy reference specimens, is that the test might not be as demanding or severe for the machine as when specimens are tested at −40 °C. More specifically, “bad” (*i.e.*, non-compliant) machines (which would not pass the verification tests at −40 °C) might successfully pass the verification at room temperature.

The parameters that effectively test the characteristics of an impact machine in a low-energy verification test are the maximum force and the rate of force application up to maximum force. If these two quantities are comparable or not statistically different between −40 °C and 21 °C for a low-energy verification lot, the objection can be rejected.

To this end, we have compared two sets of instrumented impact tests performed on a certified low-energy batch (LL-140) at −40 °C and 21 °C, in terms of maximum force *F*_m_ and rate of force application (d*F*/d*t*)_Fm_. This latter was calculated by dividing maximum force by the time to maximum force. The results, shown in Table 6, were statistically analyzed by performing a two-sample *t*-test assuming equal variances.

On the basis of the *t*-tests, the results for LL-140 are not statistically different between −40 °C and 21 °C for both maximum force (*t* = 0.251686 < *t*_crit_ = 2.0796138) and rate of force application (*t* = 1.022 < *t*_crit_ = 2.093) at a confidence level *α* = 0.05.

The objection on the presumed lower severity of room-temperature verification tests at the low-energy level can therefore be rejected.

## 6. Conclusions

The study presented in this paper demonstrated the feasibility of certifying NIST low-energy and high energy Charpy verification specimens at room temperature (21 °C ± 1 °C) instead of −40 °C.

The room-temperature tests that we conducted on five low-energy lots and four high-energy lots, tested on the three Charpy master machines located in Boulder, indicated that the variability in absorbed energy values decreased in eight out of nine cases, as demonstrated by lower coefficients of variation and lower sample sizes. The only lot for which both statistical metrics were higher at room temperature than at −40 °C was a “failed” high-energy lot, which had already proven inadequate (sample size > 5.0) during the original certification at −40 °C.

For one of the low-energy lots tested on the TK machine (the only machine with a C-shaped hammer), two specimens jammed and yielded unusually high absorbed energy values. Signs of jamming were clearly visible on the broken samples. Although the likelihood of jamming at RT appears larger than at −40 °C, as most specimens tend to remain close to the anvil/support area instead of being ejected backward, it seems unlikely that this type of behavior could be changed by modifying the heat treatment of the low-energy 4340 steel. Jamming can be clearly recognized however, and the results from a jammed specimen can be easily removed from the evaluation of a set of verification specimens.

An additional objection to the certification of room-temperature low-energy specimens (the test is less severe and demanding for the machine than at −40 °C) was disproven by statistically comparing results obtained on a low-energy batch tested at −40 °C and 21 °C. A two-sample *t*-test showed that both maximum forces and rates of force application are not statistically different at the two test temperatures.

## Figures and Tables

**Table 1:** Chemical composition of 4340 steel, weight percent.

C	Si	Mn	P	S	Mo	Ni	Cr
**0.4**	0.28	0.66	0.004	0.001	0.28	1.77	0.83

**Table 2:** Characteristics of NIST master Charpy machines.

Machine ID	Hammer weight (N)	Hammer length (mm)	Fall angle (°)	Capacity (J)	Impact speed (m/s)	Hammer type
**SI**	296.6	800.4	136.3	409.05	5.20	U
**TK**	295.3	899.1	110.7	359.52	4.89	C
**TO**	267.7	900.7	119.2	358.63	5.12	U

**Table 3:** Test matrix for the feasibility study on RT SRMs. (LL = low-energy lot; HH = high-energy lot).

Specimen lot	Number of tests on			Total tests
	SI	TK	TO	
**LL-119**		25		25

**LL-133**		30		30

**LL-138**			25	25

**LL-139**	25			

**LL-140**	25	25	25	75

**HH-136**		25		25

**HH-140**			25	25

**HH-143**	24			24

**HH-149**^a^	25	25	25	75

a “Failed” lot.

**Table 4:** Comparison between results obtained at −40 °C and 21 °C for lots tested on a single master machine. The lower values of *CV* and *n*_SS_ for each lot are highlighted in green.

Lot ID	Master machine	Test temperature (°C)	$\bar{KV}$ (J)	*σ_KV_* (J)	*CV*	*n*_SS_
						
**LL-139**	SI	*−40*	16.36	0.81	0.049	2.98
		*21*	17.91	0.56	**0.031**	**1.44**
						
**HH-143**	SI	*−40*	98.67	3.05	0.031	3.44
		*21*	106.80	3.17	**0.030**	**3.16**
						
**LL-138**	TO	*−40*	16.44	0.64	0.039	1.91
		*21*	18.75	0.41	**0.022**	**0.75**
						
**HH-140**	TO	*−40*	95.92	3.41	0.036	4.54
		*21*	103.31	2.93	**0.028**	**2.91**
						
**LL-119**	TK	*−40*	14.15	0.74	0.052	2.52
		*21*	17.10	0.60	**0.035**	**1.67**
						
**LL-133**^a^	TK	*−40*	13.98	0.83	0.060	3.19
		*21*	16.65	0.65	**0.039**	**1.96**
						
**HH-136**	TK	*−40*	78.61	2.49	0.032	2.40
		*21*	84.40	1.99	**0.024**	**2.01**

a Results obtained at room temperature after excluding the two jammed specimens (see Sec. 5.2).

**Table 5:** Comparison between results obtained at −40 °C and 21 °C for the two lots tested on the three master machines. The lower values of *CV* and *n*_SS_ for each lot are highlighted in green.

Lot ID	Test temperature (°C)	$\bar{KV}$ (J)	*σ_KV_* (J)	*CV*	*n_SS_*
					
**LL-140**	*−40*	15.98	1.26	0.079	2.75
	*21*	18.92	0.94	**0.050**	**1.46**
					
**HH-149**	*−40*	123.67	5.65	**0.046**	**7.06**
	*21*	139.29	6.85	0.049	8.50

**Table 6:** Comparison between instrumented tests conducted on LL-140 at −40 °C and 21 °C.

Test temp. (°C)	KV (J)	*F*_m_ (kN)	(d*F*/d*t*)_Fm_ (10^8^ N/s)
**−40**	18.74	33.95	2.12
	16.89	32.76	2.05
	17.84	33.81	2.11
	16.56	33.98	2.27
	17.31	31.83	1.99
	19.07	32.07	2.14
	18.80	31.92	2.00
	17.00	35.35	2.21
	17.68	33.13	2.07
	18.31	38.69	2.42
			
**Average**	**17.82**	**33.75**	**2.14**
			
**21**	19.20	32.72	2.05
	19.60	34.11	2.13
	20.03	33.95	2.00
	20.77	35.32	2.21
	19.90	34.68	2.17
	19.23	31.95	2.00
	19.80	35.42	2.08
	20.07	34.89	2.18
	20.70	34.88	2.05
	18.70	33.22	2.21
	20.03	32.37	1.90
	19.67	33.55	2.10
	19.33	33.87	1.99
			
**Average**	**19.77**	**33.92**	**2.08**

## Footnotes

1 For the explanation of sample size, please refer to Sec. 3.3, item d).

2 The NIST verification program routinely rejects lots with *n*_SS_ > 5 [3].

3 We arbitrarily assumed in this study that RT values (*CV, n*_SS_) can be considered equivalent to −40 °C values if they are less than 20 % greater.
